# Proteomic Response of *Aedes aegypti* Larvae to Silver/Silver Chloride Nanoparticles Synthesized Using *Bacillus thuringiensis* subsp. *israelensis* Metabolites

**DOI:** 10.3390/insects13070641

**Published:** 2022-07-16

**Authors:** Nantipat Chimkhan, Sutticha Na-Ranong Thammasittirong, Sittiruk Roytrakul, Sucheewin Krobthong, Anon Thammasittirong

**Affiliations:** 1 Department of Microbiology, Faculty of Liberal Arts and Science, Kasetsart University, Kamphaeng Saen Campus, Nakhon Pathom 73140, Thailand; co_enzyme_a@hotmail.com (N.C.); sutticha.n@ku.ac.th (S.N.-R.T.); 2 Microbial Biotechnology Unit, Faculty of Liberal Arts and Science, Kasetsart University, Kamphaeng Saen Campus, Nakhon Pathom 73140, Thailand; 3 Functional Ingredients and Food Innovation Research Group, National Center for Genetics Engineering and Biotechnology (BIOTEC), National Science and Technology Development Agency (NSTDA), Khlong Luang 12120, Thailand; sittiruk@biotec.or.th; 4 Center for Neuroscience, Faculty of Science, Mahidol University, Bangkok 10400, Thailand; sucheewin82@gmail.com

**Keywords:** *Aedes aegypti*, *Bacillus thuringiensis*, crystal toxin, mechanism, proteomic, silver nanoparticle

## Abstract

**Simple Summary:**

*Aedes aegypti* is a vector of important mosquito-borne diseases. Green synthesized nanoparticles (NPs) have been used to control the larvae of mosquitoes including *A. aegypti.* However, the molecular responses of *A. aegypti* larvae to green synthesized NPs remain unexplored. This work analyzed protein expression in *A. aegypti* larvae in response to treatment using green synthesized silver/silver chloride nanoparticles (Ag/AgCl NPs) based on two-dimensional polyacrylamide gel electrophoresis. Fifteen differentially expressed protein spots were selected for identification using mass spectrometry. The results showed that the six upregulated proteins in *A. aegypti* larvae responsible for green synthesized Ag/AgCl NP treatment were involved in mitochondrial dysfunction, DNA and protein damage, inhibition of cell proliferation, and cell apoptosis, implying the modes of actions of Ag/AgCl NPs. This finding has provided greater insight into the possible mechanisms of green synthesized Ag/AgCl NPs on the control of *A. aegypti* larvae.

**Abstract:**

Silver/silver chloride nanoparticles (Ag/AgCl NPs) are an alternative approach to control the larvae of *Aedes aegypti*, a vector of mosquito-borne diseases. However, the molecular mechanisms of Ag/AgCl NPs to *A. aegypti* have not been reported. In this work, Ag/AgCl NPs were synthesized using supernatant, mixed toxins from *Bacillus thuringiensis* subsp. *israelensis* (*Bti*), and heterologously expressed Cry4Aa and Cry4Ba toxins. The images from scanning electron microscopy revealed that the Ag/AgCl NPs were spherical in shape with a size range of 25–100 nm. The larvicidal activity against *A. aegypti* larvae revealed that the Ag/AgCl NPs synthesized using the supernatant of *Bti* exhibited higher toxicity (LC_50_ = 0.133 μg/mL) than the Ag/AgCl NPs synthesized using insecticidal proteins (LC_50_ = 0.148–0.217 μg/mL). The proteomic response to Ag/AgCl NPs synthesized using the supernatant of *Bti* in *A. aegypti* larvae was compared to the ddH_2_O-treated control. Two-dimensional gel electrophoresis analysis revealed 110 differentially expressed proteins, of which 15 were selected for identification using mass spectrometry. Six upregulated proteins (myosin I heavy chain, heat shock protein 70, the F_0_F_1_-type ATP synthase beta subunit, methyltransferase, protein kinase, and condensin complex subunit 3) that responded to Ag/AgCl NP treatment in *A. aegypti* were reported for NP treatments in different organisms. These results suggested that possible mechanisms of action of Ag/AgCl NPs on *A. aegypti* larvae are: mitochondrial dysfunction, DNA and protein damage, inhibition of cell proliferation, and cell apoptosis. The findings from this work provide greater insight into the action of green synthesized Ag/AgCl NPs on the control of *A. aegypti* larvae.

## 1. Introduction

*Aedes aegypti* is a vector of important mosquito-borne diseases, such as dengue, Zika, and chikungunya, which occur in tropical and subtropical regions [1]. Dengue fever is a major public health concern with approximately 100 million infections and 10,000 deaths annually in 125 countries [2]. To control dengue fever outbreaks, the World Health Organization (WHO) has recommended a combination of various approaches, such as the management of larval habitat and the application of chemical and biological insecticides [3]. However, there has been a 30-fold increase in the number of incidences over the past half-century [4] and over 6.1 billion people (about 60 percent of the global population) are predicted to be at risk of dengue in 2080 [2].

*Bacillus thuringiensis* subsp. *israelensis* (*Bti*), a mosquitocidal bacterium, produces four major δ-endotoxins (Cry4Aa, Cry4Ba, Cry11Aa, and Cyt1Aa protein) during the sporulation phase [5]. This bacterium has been used continuously in commercialized bioproducts for mosquito larvae control without any resistance reported in field populations of *Aedes* spp. [6,7,8]. Even though no resistance to *Bti* has been detected after treatment for decades, an increased tolerance has been reported to individual *Bti* toxins including Cry4Aa and Cry11Aa, in field populations of *Aedes* spp. [8]. Therefore, alternative methods need to be developed urgently to control the *A. aegypti* vector in parallel with the existing tools.

Green synthesized nanoparticles (NPs) are based on a redox reaction in which metal ions are reduced to stable NPs by organisms or their extract [9]. Various extracts of microorganisms (bacteria, fungi, algae) and higher organisms, especially plants, have been used as reducing agents for the synthesis of NPs [10,11,12,13,14,15]. The biological-based green synthesis NPs have many advantages, including being simple, rapid, non-toxic, reproducible, easy to scale up, and cost-effective, compared to chemical and physical syntheses. In addition, the biological-based NPs have been reported to be more effective, probably as a result of the attachment of biologically active components on the surface of the synthesized NPs [15,16,17]. The chemical composition of NPs, such as Ag^+^ that is released from silver nanoparticles (AgNPs), preferentially bind to a sulfhydryl (-SH) group containing proteins, leading to cytotoxicity [18]. NPs can promote the production of intracellular reactive oxygen species (ROS) that cause serious cellular damage and apoptosis [18,19]. In addition, various bioactivities of NPs have been reported, including antimicrobial, antioxidant, anti-inflammatory, anticancer, and insecticidal activities [9,10,14,17]. Silver/silver chloride nanoparticles (Ag/AgCl NPs) synthesized from various extracts of plants and microorganisms have been reported to contain high larvicidal activity against *A. aegypti* larvae [17,20,21]. Damage to the *A. aegypti* larval midgut, epithelial cells, and cortex results in physiological changes, such as shrinkage in the abdominal region, thorax shape change, and loss of lateral hairs, anal gills, and brushes, which have been reportedly caused by green synthesis NPs [22,23,24].

Even though NPs synthesized from the extracts of various organisms expressing mosquitocidal activity against *A. aegypti* larvae have been widely reported, there has been no record of response mechanisms at the molecular level of *A. aegypti* to NPs. Therefore, the aim of this work was to study the molecular response of *A. aegypti* to Ag/AgCl NPs synthesized from the metabolite of *Bti* using two-dimensional polyacrylamide gel electrophoresis (2D-PAGE) and mass spectrometric analyses to understand the mechanisms of Ag/AgCl NPs regarding mosquito larvae.

## 2. Materials and methods

### 2.1. Bacteria

The commercial strain of *Bti* was obtained from the Microbial Biotechnology Unit, Faculty of Liberal Arts and Science, Kasetsart University, Nakhon Pathom, Thailand. *Escherichia coli* harboring recombinant plasmid containing either *cry*4Aa or *cry*4Ba gene were kindly provided by the Bacterial Toxin Research Innovation Cluster (BRIC), Institute of Molecular Biosciences, Mahidol University, Nakhon Pathom, Thailand. The larvicidal activity of *Bti* and recombinant *E. coli* against two-day-old *A. aegypti* larvae was performed as described below to confirm the toxicity of the toxins before using them to construct the green synthesized Ag/AgCl NPs.

### 2.2. Synthesis of Ag/AgCl NPs Using Cry4Aa, Cry4Ba, Mixed Toxins, and Secreted Metabolites of Bti

The inclusion proteins of the mixed toxins and secreted metabolites of *Bti* were prepared according to our previous report [17]. The expression of the mixed toxins in *Bti* was analyzed using sodium dodecyl sulfate-polyacrylamide gel electrophoresis (SDS-PAGE). Single Cry4Aa and Cry4Ba toxins were prepared using heterologous expression in the *E. coli* strain JM109 under the control of the *lac*Z promoter. *E. coli* cells harboring either the *cry*4Aa or *cry*4Ba gene were grown in Luria–Bertani medium containing 100 μg/mL ampicillin at 37 °C with shaking at 100 rpm until an optical density at 600 nm (OD_600_) reached 0.3–0.5. Protein expression was induced with 0.1 mM β-isopropyl-D-thiogalactopyranoside for 4 h. After induction, the *E. coli* that overexpressed the recombinant proteins as cytoplasmic inclusions were harvested using centrifugation, resuspended in distilled water, and analyzed for protein expression using SDS-PAGE. *E. coli* strain JM109 containing the pUC12 vector was used as a negative control. Then, the harvested cells were disrupted using a sonicator (IKA Labortechnik, Staufen, Germany) at 50 W, with a cycle of 0.7, and 100% amplitude for 15 min. After sonication disruption, the inclusion protein pellets were partially purified using centrifugation at 20,000× *g* for 20 min at 4 °C, washed with phosphate buffer saline (pH 7.5), followed by three washes with cold distilled water before being kept in 2 mL deionized water. Then, the partially purified inclusion proteins from both the *Bti* and recombinant *E. coli* were solubilized (0.5 mg/mL) in 200 mL of 50 mM carbonate buffer (pH 9.5) at 37 °C for 2 h and kept at 4 °C for further processing.

The Ag/AgCl NPs were synthesized using supernatant and solubilized inclusion toxins following Thammasittirong et al. [17]. In brief, silver nitrate (AgNO_3_) was dissolved at a final concentration of 1 mM in 200 mL of supernatant or solubilized proteins and incubated under dark conditions at room temperature with shaking at 100 rpm for 72 h. The Ag/AgCl NPs were harvested using centrifugation at 20,000× *g* for 15 min, followed by three washes with sterile deionized water. The Ag/AgCl NPs were dried in an oven at 60 °C for 24 h and kept at room temperature for further analysis.

### 2.3. Characterization of Synthesized Ag/AgCl NPs

Further characterization was undertaken of the Ag/AgCl NPs synthesized using the solubilized inclusion proteins and supernatant of the commercial strain of *Bti* and the Ag/AgCl NPs synthesized using either individual Cry4Aa or Cry4Ba toxin. The morphology and composition of the synthesized NPs were examined using scanning electron microscopy (SEM; FEI Quanta-400, Brno, Czech Republic) with an operating voltage of 8 kV, equipped with energy dispersive X-ray spectrometry (EDX; Oxford Instruments, Abingdon, UK).

### 2.4. Mosquitocidal Activity Assay

Mosquitocidal activity assays were performed in two parts against *A. aegypti* larvae according to Thammasittirong et al. [17] with some modifications. In the first part, bioassay was performed to confirm the toxicity of mixed toxins from the *Bti* and individually expressed Cry4Aa or Cry4Ba toxin in *E. coli* before using them for Ag/AgCl NPs synthesis. *A. aegypti* larvae were hatched from eggs supplied by the Department of Medical Sciences, Ministry of Public Health of Thailand and reared in dechlorinated tap water supplemented with a fish diet until the larvae were aged two days. Bioassay was performed in 1 mL of different concentrations (10^2^–10^7^ cells in distilled water) of either *Bti* or *E. coli* cell suspension in 48-well plates with 10 *A. aegypti* larvae per well, with a total of 100 larvae for each concentration. Three independent replications were performed. A negative control (*E. coli* JM109 cells without recombinant plasmids) was included in each assay. After 24 h exposure, the mortality of *A. aegypti* larvae was recorded and then subjected to probit analysis to calculate the 50% and 90% lethal concentrations (LC_50_ and LC_90_, respectively), using the SPSS 16.0 software (SPSS Inc., Chicago, IL, USA). In the second part, bioassay was carried out to study the toxicity of the synthesized Ag/AgCl NPs according to Thammasittirong et al. [17]. In brief, bioassay was performed in 200 mL distilled water in a 500 mL beaker containing different concentrations of the synthesized Ag/AgCl NPs (ranging between 0.0015 and 0.3500 ppm) with 20 *A. aegypti* larvae. Three independent replications were performed. A negative control (without nanoparticles) was included in each assay. The larval mortality was recorded at 24 h after exposure and then subjected to probit analysis to calculate the LC_50_ and LC_90_ values.

### 2.5. Preparation of A. aegypti Midgut Proteins for Proteomic Analysis

Intoxication treatment for proteomic analysis was performed by treating two-day-old *A. aegypti* larvae with the Ag/AgCl NPs at the LC_25_ concentration. After 24 h treatment, the surviving larvae were cultured further for 24 h without Ag/AgCl NPs. The midgut of the surviving *A. aegypti* larvae was dissected. *A. aegypti* larvae without NP treatment were used as a negative control. Samples of 100 midguts were pooled in 100 µL of rehydration solution (8 M urea, 2% *w*/*v* CHAPS, 40 mM dithiothreitol, 0.5% *v/v* IPG buffer pH 3–10 (GE Healthcare Life Sciences, Uppsala, Sweden), 0.02% *w*/*v* bromophenol blue) containing protease inhibitors (Roche Diagnostics, Mannheim, Germany). Then, the midguts were homogenized on ice using a pellet pestle (Sigma-Aldrich, Saint Louis, MO, USA) and the extracted proteins were cleaned using a 2-D Clean-Up kit (GE Healthcare Life Sciences, Uppsala, Sweden), following the manufacturer’s instructions. Then, the protein pellets were solubilized in the rehydration solution and quantified using a 2-D Quant Kit (GE Healthcare Life Sciences, Uppsala, Sweden). The midgut protein concentration was adjusted to 50 μg in 120 μL of the rehydration solution.

### 2.6. 2D-PAGE Analysis

Two-dimension gel electrophoresis (2D-PAGE) was used to analyze the protein responses to the Ag/AgCl NP treatments. The extracted midgut proteins (50 μg in 120 μL of the rehydration solution) from the control and Ag/AgCl NP-treated *A. aegypti* larvae were used to rehydrate the 7 cm immobilized dry strips (non-linear, pH 3–10) (GE Healthcare Life Sciences, Uppsala, Sweden) for 15 h at room temperature. First-dimension electrofocusing was performed using Multiphor II isoelectric focusing system (Pharmacia Biotech, Uppsala, Sweden) at 20 °C with the following running conditions: 300 V for 1 h, 1000 V for 1 h, 2500 V for 1.5 h, and 500 V for 3 h. After complete isoelectric focusing, the focused strip gels were incubated with the first equilibration buffer (300 mM Tris-HCl, pH 8.8, 6 M urea, 30% glycerol, 2.5% SDS, 1% DTT) for 15 min and transferred to the second equilibration buffer (300 mM Tris-HCl, pH 8.8, 6 M urea, 30% glycerol, 2% SDS, 2.5% iodoacetamide) for 15 min at room temperature. The strips were washed with 1× SDS running buffer (25 mM Tris-HCl, 192 mM glycine, 0.1% (*w*/*v*) SDS) and applied onto horizontal 10% sodium dodecyl sulfate-polyacrylamide gel and then sealed with a 0.5% (*w*/*v*) agarose IEF (GE Healthcare, Uppsala, Sweden) in the SDS running buffer. Next, the proteins were separated according to their molecular weight on the SDS-PAGE using the Bio-Rad Mini Protean II gel electrophoresis system (Bio-Rad, Hercules, CA, USA) at 50 V for 15 min and then at 200 V for 45 min. Gels were stained using a PageSilver silver staining kit (Fermentas, Baden-Wurttemberg, Germany) according to the manufacturer’s directions. Three independent experiments were performed.

### 2.7. In-Gel Digestion and Protein Identification Using LC-MS/MS Analysis

Protein spots were selected and manually excised from the silver-stained 2D gel using a razor blade. Each spot gel was sliced into 1 mm^3^ pieces and dehydrated twice with acetonitrile (5 min each time). Proteins in the gel pieces were reduced with 10 mM DTT in 10 mM ammonium bicarbonate at 56 °C for 1 h and then alkylated with 100 mM iodoacetamide in 10 mM ammonium bicarbonate at room temperature for 1 h. The gels were twice dehydrated with acetonitrile for 5 min and dried using a speed vacuum (ThermoSavant, Holbrook, NY, USA). Digestion was performed by soaking the gels in sequencing-grade trypsin solution (50 ng) for 6 h at 37 °C. The peptides were extracted using 30 µL of 0.1% (*v*/*v*) formic acid in 50% (*v*/*v*) acetonitrile, with shaking for 10 min. The extracts containing tryptic peptide were dried using speed vacuum and redissolved in 10 µL of 0.1% (*v*/*v*) formic acid. The extracted peptides were further analyzed using an Ultimate3000 Nano/Capillary LC System (Dionex, Camberley, UK) coupled to a Hybrid quadrupole Q-TOF impact II™ (Bruker Daltonics GmbH, Bremen, Germany) equipped with a nano-captive spray ion source. Samples (1 µL) were injected into the trapping column (300 μm id × 5 mm, C18 PepMap100, 5 μm; Thermo Scientific, Dreieich, Germany) and resolved on a separating column (PepSwift Monolithic Nano Column, 100 µm × 5 cm; Dionex, Darmstadt, Germany). Peptides were separated and eluted from the column using a linear gradient at a flow rate of 1 µL/min for the mobile phase A (0.1% formic acid in water) and the mobile phase B (0.1% formic acid in 80% acetonitrile). Mass spectrometry was performed in the positive-ion mode and scanned in the range *m*/*z* 150–2200 (Bruker Daltonics, Bremen, Germany). The obtained MS/MS data were analyzed using the MASCOT software (Matrix Science, London, UK) against the *A. aegypti* database. The MASCOT search criteria used were trypsin digestion with one missed cleavage allowed, carbamidomethylation (C) as fixed modification, deamidated (NQ), oxidation (M), phospho (ST), and phospho (Y) as the variable modification.

## 3. Results and Discussion

### 3.1. Mosquitocidal Activity of Bti Toxins

**Figure 1:**
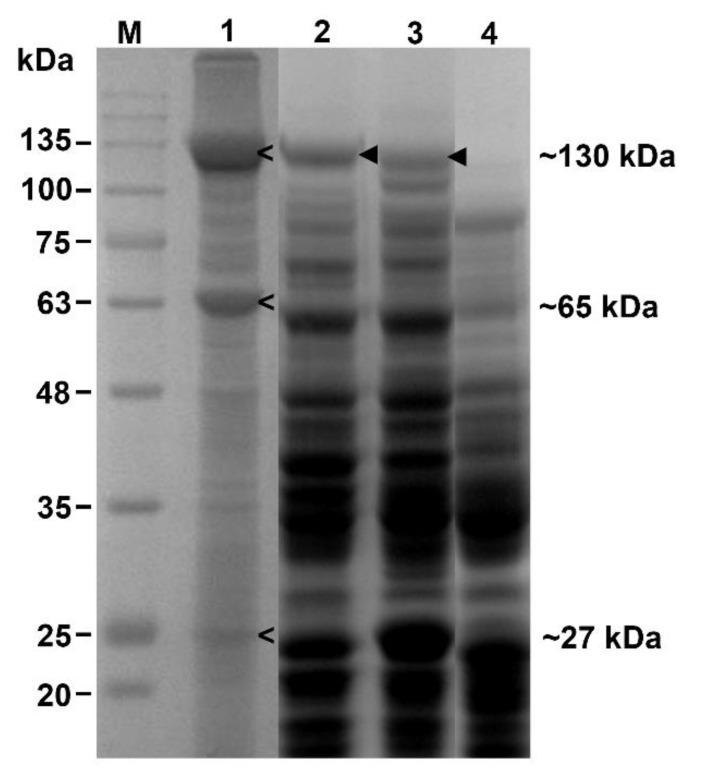
Expression of *B. thuringiensis* subsp. *israelensis* (*Bti*) toxins analyzed using SDS-PAGE (10% gel). Lane M: protein standard marker; lane 1: protein expression of *Bti*; lane 2: protein expression of *E. coli* harboring *cry*4Ba gene; lane 3: protein expression of *E. coli* harboring *cry*4Aa gene; lane 4: protein expression of *E. coli* JM109 harboring pUC12 (negative control). Whole protein lysate was extracted from ~10^7^ cells. Arrows (**<**) indicate expression of Cry toxins in *Bti*; ◄ indicate heterologous expression of Cry toxins in *E. coli*.

*Bti* produces at least four mosquitocidal toxins (Cry4Aa, Cry4Ba, Cry11Aa, and Cyt1Aa) accumulated as protein inclusions in cells [5]. In the current study, the commercial strain of *Bti* and the recombinant strain of *E. coli* expressing either Cry4Aa or Cry4Ba were assayed for mosquitocidal activity against two-day-old *A. aegypti* larvae prior to using them for Ag/AgCl NPs synthesis. SDS-PAGE showed the expression of mixed toxins in *Bti*, including Cry4Aa and Cry4Ba proteins at approximately 130-kDa, Cry11Aa protein at approximately 65-kDa, Cyt1Aa at approximately 27-kDa (Figure 1, lane 1), and individual heterologously expressed Cry4Ba or Cry4Aa in *E. coli* (Figure 1, lanes 2 and 3, respectively). The mortality was recorded at 24 h after exposure to the toxin. The LC_50_ values analyzed using probit analysis are shown in Table 1. The results showed that the *E. coli* expressed Cry4Aa or Cry4Ba protein exhibited lower mosquitocidal activity than that of *Bti* containing a mixture of toxins that resulted in a synergistic effect, as reported by Khasdan et al. [25] and Lailak et al. [26]. In addition, the higher toxicity of *Bti* might have been due to the higher expression of the toxins in *Bti* than in the heterologous host. The toxicity of the commercial strain of *Bti* in this work was higher than reported by Lobo et al. [27]. The lower larvicidal activity against *A. aegypti* larvae of the Cry4Aa toxin is usually observed compared to the Cry4Ba toxin [28]. However, the bioassay results showed that the mixed toxins from *Bti* and the individually expressed Cry4Aa and Cry4Ba toxins from *E. coli* exhibited larvicidal activity against *A. aegypti* larvae; thus, they could be used for further NPs synthesis.

### 3.2. Synthesis and Characterization of Ag/AgCl NPs

**Figure 2:**
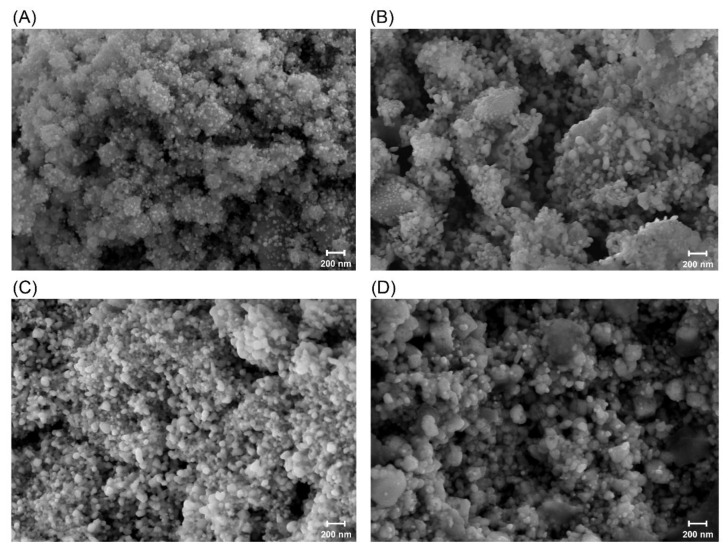
SEM images of silver/silver chloride nanoparticles (Ag/AgCl NPs) synthesized using (**A**) supernatant of *Bti*; (**B**) solubilized inclusion proteins of *Bti* (mix toxins); (**C**) solubilized Cry4Aa protein; (**D**) solubilized Cry4Ba protein.

**Figure 3:**
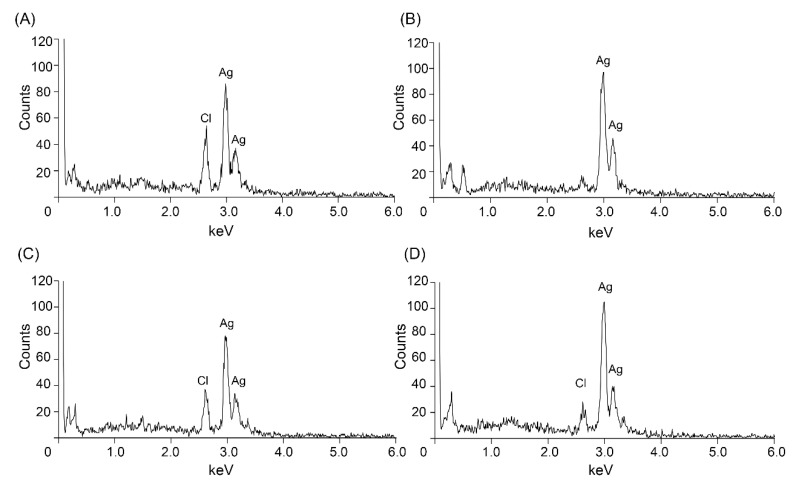
Energy-dispersive X-ray spectrometry spectra of Ag/AgCl NPs synthesized using (**A**) supernatant of *Bti*; (**B**) solubilized inclusion proteins of *Bti* (mix toxins); (**C**) solubilized Cry4Aa protein; (**D**) solubilized Cry4Ba protein.

Ag/AgCl NPs were synthesized using the supernatant and solubilized inclusion proteins from *Bti* and also synthesized from solubilized Cry4Aa and Cry4Ba toxins that were heterologously expressed in *E. coli*. The SEM results revealed that the NPs were mostly spherical in shape with a size range of 25–100 nm (Figure 2). Most green synthesized NPs have been reported as spherical in shape with various sizes, as reviewed in Koduru et al. [12]. It was observed that the NPs synthesized from the *Bti* supernatant (Figure 2A) were smaller than those synthesized from the solubilized inclusions of mixed and individual toxins (Figure 2B–D). Energy-dispersive X-ray spectrometry revealed a strong signal of silver at approximately 3 keV (Figure 3A–D) that was typical for the absorption of metallic AgNPs [29], which confirmed the presence of the element Ag in the synthesized NPs. This 3 keV signal peak was also reported in various green synthesized NPs [13,20,30]. In addition, a chloride signal at approximately 2.7 keV was observed in the NPs synthesized using the supernatant of *Bti* and solubilized Cry4Aa and Cry4Ba proteins (Figure 3A, C, and D, respectively). These results implied that the silver chloride (AgCl) NPs were also constructed with these conditions. The formation of AgCl NPs is usually reported together with AgNPs when NPs are constructed using supernatant containing Cl^-^ or plant extract as a reducing agent [31,32].

### 3.3. Toxicity of Ag/AgCl NPs

The toxicity levels of Ag/AgCl NPs synthesized using the supernatant, mixed toxins, and individual toxins of *Bti* were assayed against two-day-old *A. aegypti* larvae. The bioassay results revealed that the highest larvicidal activity (LC_50_ 0.133 ppm, LC_90_ 0.292 ppm) was obtained from the Ag/AgCl NPs synthesized using the supernatant of *Bti* (Table 2). The toxicity against *A. aegypti* was close to that reported for AgNPs synthesized using the supernatant of *Bacillus thuringiensis* (LC_50_ 0.1 ppm, LC_90_ 0.39 ppm) by Banu et al. [33]. Interestingly, the toxicity of NPs synthesized from the mixed toxins (solubilized inclusion proteins of *Bti*) was lower than that of the single Cry4A toxin and was close to the toxicity of the single Cry4Ba toxin (Table 2). These results indicated that the toxicity of green synthesized Ag/AgCl NPs was not correlated with the toxicity of the reducing agents used for the NP synthesis. The difference in mosquitocidal activity may have been due to the differences in Ag/AgCl NP properties, such as size and shape. However, our previous report revealed that the mosquitocidal toxicity of green synthesis AgNPs was higher than that of chemically synthesized NPs [17].

### 3.4. Analysis of Molecular Response of A. aegypti to Ag/AgCl NP Treatment

**Figure 4:**
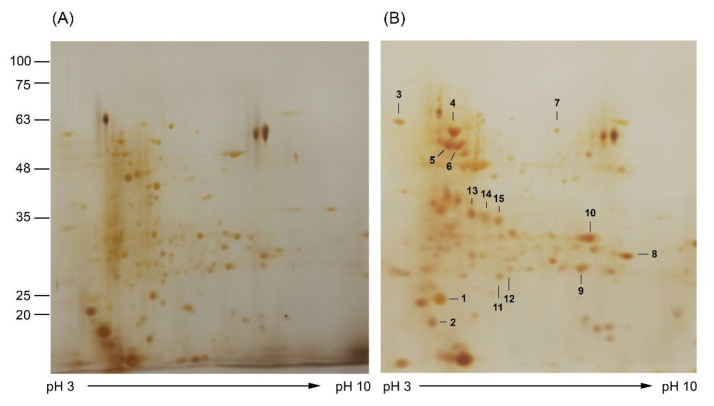
Silver-stained 2D gel images of midgut proteins: (**A**) control group treated with ddH_2_O; (**B**) group treated with concentration at LC_25_ of Ag/AgCl NPs synthesized using supernatant of *Bti*. Numbered spots are differentially expressed proteins that were selected for identification using LC-MS. Positions of molecular size markers (in kDa) are indicated on the left and the pH range of isoelectric focusing on the bottom.

The cytotoxicity of NPs has been intensively studied and reported in various cell types [34,35]. AgNPs were reported to interact with the cell membrane and activated signaling pathway to generate ROS, leading to damage of DNA and proteins, inhibition of cell proliferation, and subsequent cell apoptosis [36]. In addition, mitochondrial dysfunction has been reported as one of the effects of AgNPs [37]. To our knowledge, there has been no report on the molecular response of *A. aegypti* larvae to the green synthesized Ag/AgCl NP treatment. Therefore, in the current study, we used a proteomic approach to study the protein expression response against Ag/AgCl NP exposure. The Ag/AgCl NPs synthesized using the supernatant of *Bti* containing the highest mosquitocidal activity were selected for *A. aegypti* treatment. After intoxication treated with the LC_25_ concentration of Ag/AgCl NPs, midgut proteins were extracted and analyzed using 2D-PAGE. The results revealed different expression levels of the midgut proteins after exposure to the Ag/AgCl NPs compared with the untreated (ddH_2_O) control (Figure 4). The 2-DE gels revealed 110 protein spots with molecular weights lower than 75 kDa (Figure 4). These results were similar to the findings of Popova-Butler and Dean [38] who reported that only *A. aegypti* midgut proteins with sizes less than 75 kDa were observed on the 2-DE gel. In total, 15 differentially expressed proteins, of which 14 were upregulated and 1 was downregulated (Figure 4), were selected, excised from the 2-DE gel, and subjected to in-gel digestion followed by identification using LC-MS/MS. The identified proteins, shown in Table 3, were classified into eight groups according to biological function, consisting of the cytoskeleton, stress response, energy production, transcription, signal transduction, protease activity, kinase activity, and membrane-bound ion channel. Some of these have been widely reported to be associated with the cellular response to NP treatment, such as myosin I heavy chain (spot 1), heat shock protein 70 (spot 3,12), F_0_F_1_-type ATP synthase beta subunit (spot 6), methyltransferase (spot 14), and condensin complex subunit 3 (spot 5). The position of protein spots, such as myosin I heavy chain isoform X1 (spot 1), heat shock 70 (spot 3), and condensin complex subunit 3 (spot 5), were lower than the predicted mass. This may have been due to fragmentation during the purification process, as reported in the preparation of brush border membrane vesicle (BBMV) proteins from the *A. aegypti* midgut [38,39].

The myosin I heavy chain is an ATP-dependent motor protein. Class I myosin and actin have been reported to be involved in the cellular uptake and intracellular trafficking of NPs [40]. In addition, it was incorporated with actin to generate membrane invagination, coat-pit formation, conscription, and vesicle scission in the process of endocytosis [40]. Myosin I heavy chain increased in abundance after treatment of mussels (*Mytilus galloprovincialis*) with AgNPs and silver ions (Ag^+^) [41]. In addition, its expression level was altered when treating the human hepatoma cell line HepG2 with AgNPs and Ag^+^ [19]. Upregulation of the myosin I heavy chain in the current study (spot 1) may be related to the uptake and trafficking of Ag/AgCl NPs in midgut epithelial cells. In contrast, other studies reported no detectable change in expression of the myosin I heavy chain in the *A. aegypti* midgut after exposure to the Cry4Ba and Cry11Aa toxins [39,42], suggesting that a different mode of action for larvicidal activity is involved by which Cry toxins create pores on midgut epithelial cells, while the AgNPs and Ag^+^ can be taken up into the cells without pore formation.

NPs have been reported to influence the secondary structure of proteins, either by disturbing the normal folding or causing indirect damage to proteins, thus leading to the possible loss of biological function [41,43]. Hsp70, a 70-kDa heat shock protein, is involved in the folding assembly of newly synthesized protein, the refolding of misfolded and aggregated proteins, and the membrane translocation of secretory and organellar proteins [44]. In addition, Hsp70 is implicated in the cellular stress response process, which is inducible by many stressful stimuli, including heat, cold, UV radiation, toxic substances such as insecticides and heavy metals, and also by NPs [45,46,47,48]. In addition to the effect on the structure and function of proteins, cell apoptosis has been reported as one effect of AgNPs [49,50]. Heat shock proteins (HSPs) have been reported to be involved in protecting cells from apoptosis by preventing the activation of caspase in the cell death pathway [51]. The incremental expression of Hsp70 in our results (spot 3) may have prevented the misfolded midgut proteins caused by conformational changes or may have inhibited apoptotic machinery. Downregulation of the Hsp90 protein in the *A. aegypti* midgut was found after exposure to *B. thuringiensis* Cry11Aa toxin [42], perhaps due to the different mode of action by which Cry toxins create pores on midgut epithelial cells but do not affect the structure and function of cellular protein.

A reduction in adenosine triphosphate (ATP) production due to mitochondrial dysfunction is one process that is affected by NPs [52,53]. The F_0_F_1_-type ATP synthase beta subunit is involved in ATP synthesis using the proton gradient across the mitochondrial membrane by the respiratory chain [54]. In addition to being located on the mitochondrial inner membrane, the F_0_F_1_-type ATP synthase is located on the surfaces of many cells, including endothelial cells, adipocytes, keratinocytes, hepatic cells, and the *Aedes albopictus* C6/36 cell line [55]. While ATP synthase on the mitochondrial membrane has a function in ATP synthesis, the cell surface ATP synthase has been implicated in various cellular activities, such as mediation of cellular pH, response to antiangiogenic agents, and cholesterol homeostasis [56]. Upregulation of the F-type ATP synthase protein in the current study (spot 6) was similar to various organisms after NP treatment [41,57,58,59]. The *A. aegypti* midgut cells may require high F_0_F_1_-type ATP synthase activity to compensate for the decrease in ATP production caused by NPs. In addition, the higher ATP level may activate the defense mechanisms response to stress conditions, similar to the upregulation of F_0_F_1_ type ATP synthase reported in *A. aegypti* larvae treated with Cry11Aa toxin [42].

DNA methylation changes, including hyper- and hypo-methylation, have been reported in response to NPs [34,39,60,61]. Hypomethylation of DNA activates transcription, whereas gene silencing is a result of DNA hypermethylation [35]. Hypermethylation of chromatin by methyl transferase activity has been suggested as being involved in controlling access to the damaged regions of DNA by repair signaling proteins [62]. Upregulated expression of methyltransferase in the current study (spot 14) was suggested for hypermethylation DNA, which is a signal for DNA repair. In addition, upregulation of protein kinase was observed in the current study (spot 15). Kinase is responsible for cellular transduction signaling [63]. It was upregulated in several cell types in response to AgNP treatment [64,65]. The mitogen-activated protein kinase of the stress signaling network was suggested as a response to the defense of AgNP exposure in *Caenorhabditis elegans* [66]. The extracellular signal-regulated kinase, which was an early response to AgNP treatment, is involved in signaling for DNA repair [67]. Upregulated expression of kinase in the current study could be related to the phosphorylation of the signal proteins’ response to repairing the DNA damaged by oxidative stress induced by Ag/AgCl NPs.

Chromosome missegregation during mitosis has been reported to arrest the cell cycle as an effect following exposure to AgNPs [68]. Condensin is a hetero-pentameric protein complex that is essential for chromosome organization, such as mitotic chromosome assembly and segregation, and DNA recombination and repair processes [69,70]. Condensin II was reported to function in reducing DNA damage by maintaining the genomic stability in *Arabidopsis thaliana* exposed to excess boron [70]. Condensin complex subunit III was overexpressed in human keratinocytes after exposure to titanium dioxide NPs [60]. Upregulated expression of the condensin protein in the current study (spot 5) may be involved in the physiological protection of the genome from genotoxic substances and/or repairing DNA double-strand breaks and damaged replication forks as the possible mechanisms of condensin II, as suggested by Sakamoto et al. [70].

## 4. Conclusions

We constructed and characterized Ag/AgCl NPs synthesized using supernatant, mixed toxins from *Bti*, and heterologously expressed Cry4Aa and Cry4Ba toxins. The Ag/AgCl NPs synthesized using the supernatant of *Bti* containing the highest mosquitocidal activity were selected to study the molecular response of *A. aegypti* midgut cells. In total, 110 differentially expressed protein spots were observed, of which 15 were selected for identification using mass spectrometry. Myosin I heavy chain, heat shock protein 70, the F_0_F_1_-type ATP synthase beta subunit, methyltransferase, protein kinase, and condensin complex subunit 3, whose expression is modulated in response to Ag/AgCl NP treatments, are involved in mitochondrial dysfunction, DNA and protein damage, inhibition of cell proliferation, and apoptosis. These findings provided the molecular response of *A. aegypti* larvae to green synthesized Ag/AgCl NPs, suggesting the possible mechanisms of action of Ag/AgCl NPs in *A. aegypti* larvae.

## Figures and Tables

**Table 1:** Mosquito larvicidal activity of *B. thuringiensis* subsp. *israelensis* and *E. coli* expressing different Cry toxins against *A. aegypti* larvae.

Bacterium	LC_50_ (Cells/mL)
*Bacillus thuringiensis* subsp. *israelensis*	4.62 × 10^2^
*Escherichia coli* + Cry4Aa	1.20 × 10^6^
*Escherichia coli* + Cry4Ba	6.20 × 10^5^
*Escherichia coli* JM109 + pUC12	-

**Table 2:** Mosquitocidal activity of Ag/AgCl NPs synthesized using supernatant and solubilized inclusion proteins of *Bti* against *A. aegypti* larvae.

Source of AgNPs	LC_50_ (µg/mL) (LCL-UCL)	LC_90_ (µg/mL) (LCL-UCL)
Supernatant of *Bti*	0.133 (0.089–0.194)	0.292 (0.222–0.446)
Inclusion proteins of *Bti*	0.206 (0.161–0.273)	0.423 (0.338–0.591)
Cry4Aa protein	0.148 (0.018–0.508)	0.358 (0.219–2.020)
Cry4Ba protein	0.217 (0.137–0.423)	0.415 (0.286–0.985)

LCL: lower confidence limits; UCL: upper confidence limits.

**Table 3:** Identification of differentially regulated proteins between control (ddH_2_O) and Ag/AgCl NP treatment groups. Database searches using the Mascot program against *A. aegypti* protein database.

Spot Number ^a^	Top Match Protein	Accession Number ^b^	Predicted Mass (kDa)	Peptide Match	Expression ^c^
**Cytoskeleton protein**					
1	Myosin I heavy chain isoform X1	1218220707	276.3	14	↑
9	Tau and MAP protein, tubulin-binding repeat	108871405	31.7	9	↑
13	Microtubule-associated protein futsch-like isoform X1	1218209813	150.4	9	↑
**Stress response protein**					
3	Heat shock 70-kDa protein 4 isoform X1	1218258666	94.0	9	↑
12	Heat shock 70-kDa protein 4 isoform X2	1218258668	91.1	9	↑
**Energy production protein**					
6	F0F1-type ATP synthase beta subunit	94468834	54.0	12	↑
**Transcriptional processing associated protein**					
5	Condensin complex subunit 3	1218229275	170.5	9	↑
14	Methyltransferase	108880940	113.2	9	↑
**Signal transduction protein**					
4	Zinc finger C2HC domain-containing protein 1C isoform X1	1218219689	58.8	9	↑
**Protein with protease activity**					
11	Lectizyme	157124382	31.4	8	↑
**Protein modification**					
15	Protein tyrosine kinases	403182426	123.5	9	↑
**Membrane bound ion channel protein**					
8	Amiloride-sensitive sodium channel	108869271	54.0	9	↑
**Uncharacterized protein**					
2	Putative leucine rich repeat some	943361693	110.3	9	↓
10	Uncharacterized protein	1218249106	86.8	9	↑
7	Serine-rich protein	108879398	62.7	9	↑

^a^ Spot number as shown in Figure 4; ^b^ protein in the NCBI database for which significant peptide mass matches; ^c^ upregulated expression “↑”; downregulated expression “↓”.

## Data Availability

The data presented in this study are available on request from the corresponding author.
